# Exploring gut microbiota and metabolite alterations in patients with thyroid-associated ophthalmopathy using high-throughput sequencing and untargeted metabolomics

**DOI:** 10.3389/fendo.2024.1413890

**Published:** 2024-07-29

**Authors:** Xiran Zhang, Kui Dong, Xinxin Zhang, Zhiming Kang, Bin Sun

**Affiliations:** ^1^ Laboratory of Ophthalmic Microbiology, Shanxi Eye Hospital, Taiyuan, China; ^2^ Shanxi Province Key Laboratory of Ophthalmology, Shanxi Eye Hospital, Taiyuan, China

**Keywords:** thyroid-associated ophthalmopathy, intestinal flora, metabolite alterations, high throughput sequencing, untargeted metabolomics

## Abstract

**Introduction:**

Thyroid-associated ophthalmopathy (TAO) is an autoimmune-driven orbital inflammatory disease. Despite research efforts, its exact pathogenesis remains unclear. This study aimed to characterize the intestinal flora and metabolic changes in patients with TAO to identify the flora and metabolites associated with disease development.

**Methods:**

Thirty patients with TAO and 29 healthy controls were included in the study. The intestinal flora and metabolites were analyzed using high-throughput sequencing of the 16S rRNA gene and non-targeted metabolomics technology, respectively. Fresh fecal samples were collected from both populations for analysis.

**Results:**

Reduced gut richness and diversity were observed in patients with TAO. Compared to healthy controls, significant differences in relative abundance were observed in patients with TAO at the order level *Clostridiales*, family level *Staphylococcaceae*, genus level *Staphylococcus*, *Fournierella*, *Eubacterium siraeum*, *CAG-56*, *Ruminococcus gnavus*, *Intestinibacter*, *Actinomyces*, and *Erysipelotrichaceae UCG-003* (logFC>1 and P<0.05). *Veillonella* and *Megamonas* were closely associated with clinical symptoms in patients with TAO. Among the 184 significantly different metabolites, 63 were upregulated, and 121 were downregulated in patients with TAO compared to healthy controls. The biosynthesis of unsaturated fatty acids was the significantly enriched metabolic pathway. Correlation analysis revealed *Actinomyces* was positively correlated with NAGlySer 15:0/16:0, FAHFA 3:0/20:0, and Lignoceric Acid, while *Ruminococcus gnavu* was positively correlated with Cer 18:0;2O/16:0; (3OH) and ST 24:1;O4/18:2.

**Conclusion:**

Specific intestinal flora and metabolites are closely associated with TAO development. Further investigation into the functional associations between these flora and metabolites will enhance our understanding of TAO pathogenesis.

## 1 Introduction

Thyroid-associated ophthalmopathy (TAO) is an orbital inflammatory disease caused by autoimmune reactions. Despite extensive research, the precise pathogenesis of TAO remains unknown, although it is widely postulated to stem from shared antigens, such as the thyroid-stimulating hormone receptor (TSHR) and insulin growth factor 1 receptor (IGF-1R), expressed in periorbital tissues. TAO unfolds as a predominantly cellular autoimmune disorder, accompanied by humoral immune responses, culminating in pathological changes such as lymphocytic infiltration, periorbital tissue edema, myofibroblast differentiation, and adipocytosis. These pathological alterations collectively contribute to the clinical manifestations of exophthalmos and extraocular muscle dysfunction (1). The etiology of TAO is multifaceted, influenced not only by external environment such as smoking, radioactive iodine treatment, and selenium deficiency but also by internal factors such as immune cell abnormalities, autoantibodies, and dysbiosis of intestinal flora. Dysbiosis has sparked significant scholarly interest as it triggers cascading immune responses, becoming a focal point of investigation (2–4).

In recent years, the spotlight in the medical field has increasingly turned towards the intricate world of intestinal flora – the diverse microbial communities residing in the human intestinal tract. These communities play important roles in digestion, metabolism, and immune regulation, making them a focal point of research due to their implications for various diseases. Particularly noteworthy is their emerging connection to ocular diseases, such as uveitis, age-related macular degeneration, glaucoma, and dry eye. This link is elucidated by the intestinal-ocular axis hypothesis, proposing that dysbiosis of intestinal flora can induce local and systemic immune responses, thereby contributing to the development of ocular diseases (5, 6). In the context of TAO, previous research has indicated a potential role for intestinal flora in modulating immune responses. Studies have revealed that alterations in the composition, distribution, and metabolites of intestinal flora can impact immune cells and cytokines, influencing the immune landscape associated with TAO. For instance, dysbiosis of intestinal flora may prompt intestinal dendritic cells to secrete transforming growth factor beta (TGF-β), thereby disturbing the balance between Th17 and Treg cells, which has been implicated in the pathogenesis of TAO (7, 8).

While previous studies have underscored the significance of metabolites derived from various sources such as orbital fat, tears, blood, and urine samples in TAO development (9–11), the role of intestinal metabolites resulting from intestinal flora activity remains largely unexplored. Therefore, this study aimed to unravel the distinct characteristics of intestinal flora and metabolic changes in patients with TAO, with a specific focus on identifying the microbial composition and metabolites closely linked to disease development. By shedding light on these factors, our goal was to offer novel perspectives that could potentially inform more effective strategies for the treatment and prevention of TAO.

Thus, a joint investigation of intestinal flora and intestinal metabolites of patients with TAO using high-throughput sequencing of the 16S rRNA gene and untargeted metabolomics holds promise for providing new insights into the pathogenesis of TAO.

## 2 Materials and methods

### 2.1 Study population

Thirty patients with TAO (21 males and 9 females) were included in the study, all of whom were receiving care at Shanxi Provincial Eye Hospital between July 2022 and July 2023. Additionally, 29 healthy volunteers were recruited to serve as the control group. Before enrollment, thyroid function in TAO patients was controlled, and patients had not taken anti-thyroid medications. Diagnosis of TAO was based on Bartley’s diagnostic criteria, and clinical assessments were conducted by the same ophthalmologist. The evaluation included the examination of clinical symptoms such as eyelid recession and exophthalmos, as well as the assessment of disease activity using the TAO Clinical Activity Score (CAS) and severity based on the European Guidelines for Graves’ Ophthalmopathy (EUGOGO) (12, 13). Exclusion criteria encompassed systemic conditions (diabetes mellitus, hypertension, infectious diseases, blood disorders, and autoimmune diseases), gastrointestinal factors (acute enteritis within the last 6 months, history of blood in the stool or constipation within the last 3 months, and history of gastrointestinal surgery), and medication usage (laxatives, prebiotics, probiotics, herbal, or antibiotic treatments in the last 6 months, glucocorticoid therapy, immunosuppressive drugs, targeted drugs, and radioiodine therapy within the last 6 months). Healthy controls underwent a thorough medical history review and underwent the same exclusion criteria as patients with TAO. Additionally, thyroid function tests (FT3, FT4, TSH, TPOAb, TGAb, and TRAb) were performed to exclude controls with abnormal thyroid function. Dietary habits and lifestyle habits were recorded simultaneously in both groups.

The study protocol was approved by the Hospital Ethics Committee, and all participants provided written informed consent in accordance with the principles outlined in the Declaration of Helsinki (Ethical approval No. SXYYLL-KSSC004).

### 2.2 Stool and blood sample collection

Fresh fecal samples were collected from participants following an overnight fast (8 hours). The middle part of each fecal sample was isolated using a sterile spoon in the laboratory. Fecal samples designated for intestinal flora sequencing were stored in 50 mL EP tubes, with 2 g of fecal material per tube, while those for the intestinal flora metabolism analysis were stored in 5 mL EP tubes, with 250 mg of fecal material per tube. The samples were then divided into portions and promptly submerged in liquid nitrogen for 15 min before being transferred to a -80 °C freezer to avoid repeated freezing and thawing. In the healthy group, 4 mL of blood was collected and placed in tubes with yellow caps for thyroid function assays.

### 2.3 16S rRNA gene sequencing analysis

A library of small fragments of the V3-V4 region of the 16S rRNA gene was constructed using the DNA processing method outlined in Qiagen 51604. Subsequently, double-end sequencing was performed using the Illumina NovaSeq sequencing platform. Amplicon sequence variants (ASVs) were obtained through read splicing filtering and DADA2 noise reduction. Species annotation and abundance analysis were performed to determine the species composition of the samples (14). α-diversity and β-diversity analyses were utilized to reveal differences in community structure, while the LEfSe method was used to discriminate taxonomic types. Species with significant intergroup differences were screened using the metagenomeSeq method, applying |logFC|>1 and P<0.05 as the difference significance screening threshold (15, 16). Finally, the correlation between the gut flora and TAO clinical indicators was analyzed using Spearman’s correlation coefficient.

### 2.4 Non-targeted fecal metabolome LC-MS analysis

Fecal samples weighing 100 mg were pulverized using liquid nitrogen and treated with 500 μL of 80% methanol solution, followed by vortex shaking. After centrifugation at 4°C and 15000 g for 20 min on ice, the supernatant was collected, diluted to a methanol concentration of 53%, centrifuged again, and subjected to LC-MS analysis (17). Raw data files were imported into CD3.3 for processing to obtain metabolite identification and relative quantification. The identified metabolites were annotated using the KEGG database. In the multivariate statistical analysis, partial least squares discriminant analysis (PLS-DA) was performed to obtain Variable Importance in Projection (VIP) values for each metabolite. For the univariate analysis, significantly different metabolites were determined based on thresholds of VIP>1.0, Fold Change (FC) >1.2, or FC<0.833 with P<0.05. Matchstick plots were generated based on different metabolites obtained from each group comparison combination using the KEGG database to perform pathway enrichment analysis. Metabolic pathways were considered enriched when the ratio of x/n to y/n was greater than 1, and significant enrichment was determined when the pathway’s P-value was <0.05 (18–20). Differential metabolites from significantly enriched pathways were evaluated as potential biomarkers using Receiver Operating Characteristic (ROC) curves.

### 2.5 Cross-homology correlation network analysis

Pearson’s statistic was employed to calculate correlation coefficients between the relative abundance of each genus at the genus level and the quantitative values of different differential metabolites in patients with TAO. Subsequently, a network diagram of the correlation analysis was generated using R3.5.0.

### 2.6 Statistical analysis

Basic information of patients with TAO and the healthy group was analyzed using SPSS version 26.0, with statistical significance set at P < 0.05. Measurement data following a normal distribution were expressed as mean ± standard deviation, and comparisons were made using independent samples t-test. For measurement data not following a normal distribution, the median (interquartile range) was used, and comparisons were conducted using the Mann-Whitney U test. Comparison of count data composition between groups was performed using the chi-square test. Additional statistical methods are described in the bioinformatics analysis process described above.

## 3 Results

### 3.1 Analysis of basic information

Thirty patients with TAO and 29 healthy controls were recruited according to the predetermined inclusion and exclusion criteria. The characteristics of all enrolled volunteers are shown in **Table 1**. No significant differences were observed between the two groups in terms of sex, age, or body mass index (BMI). There were no differences between the two groups in some of their dietary habits and lifestyle (**Table 2**).

**Table 1:** Comparison of general information between TAO and the healthy control group.

	Total number of case	Sex (male/female)	Age (year)	BMI(kg/m^2^)
TAO group	30	21/9	43.40 ± 10.38	23.84 ± 3.11
Control group	29	14/15	41.79 ± 7.61	24.84 ± 3.67
t/x^2^	–	2.884	0.676	-1.131
p	–	0.089	0.502	0.263

**Table 2:** Comparison of dietary habits and lifestyles between two groups.

		TAO group(case,%)	Control group(case,%)	OR value	95% CI	X^2^ value	P value
Dietary habits							
meats	Somet-imes	16 (53.3)	19 (65.5)	1.0			
	often	14 (46.7)	10 (34.5)	0.602	0.211-1.718	0.907	0.341
fruits	Somet-imes	21 (70.0)	16 (55.2)	1.0			
	often	9 (30.0)	13 (44.8)	1.896	0.650-5.528	1.386	0.239
carbohydrates (e.g., semolina, potatoes, bread, cakes)	Somet-imes	15 (50.0)	10 (34.5)	1.0			
	often	15 (50.0)	19 (65.5)	1.900	0.666-5.419	1.454	0.228
desserts	Somet-imes	5 (16.7)	6 (20.7)	1.0			
	often	25 (83.3)	23 (79.3)	0.767	0.206-2.856	0.157	0.692
flavor(spicy)	Yes	12 (40.0)	10 (34.5)	1.0			
	No	18 (60.0)	19 (65.5)	1.267	0.440-3.650	0.192	0.661
midnight snack	Yes	8 (26.7)	5 (17.2)	1.0			
	No	22 (73.3)	24 (82.8)	1.745	0.496-6.143	0.763	0.383
strong tea, coffee	Yes	5 (16.7)	7 (24.1)	1.0			
	No	25 (83.3)	22 (75.9)	0.629	0.174-2.267	0.508	0.476
Lifestyle							
go to bed (> 22:00)	Yes	22 (73.3)	23 (79.3)	1.0			
	No	8 (26.7)	6 (20.7)	0.717	0.214-2.404	0.291	0.590

### 3.2 Fecal high-throughput sequencing results in patients with TAO patients and healthy controls

#### 3.2.1 Analysis of intestinal flora diversity

**Figure 1:**
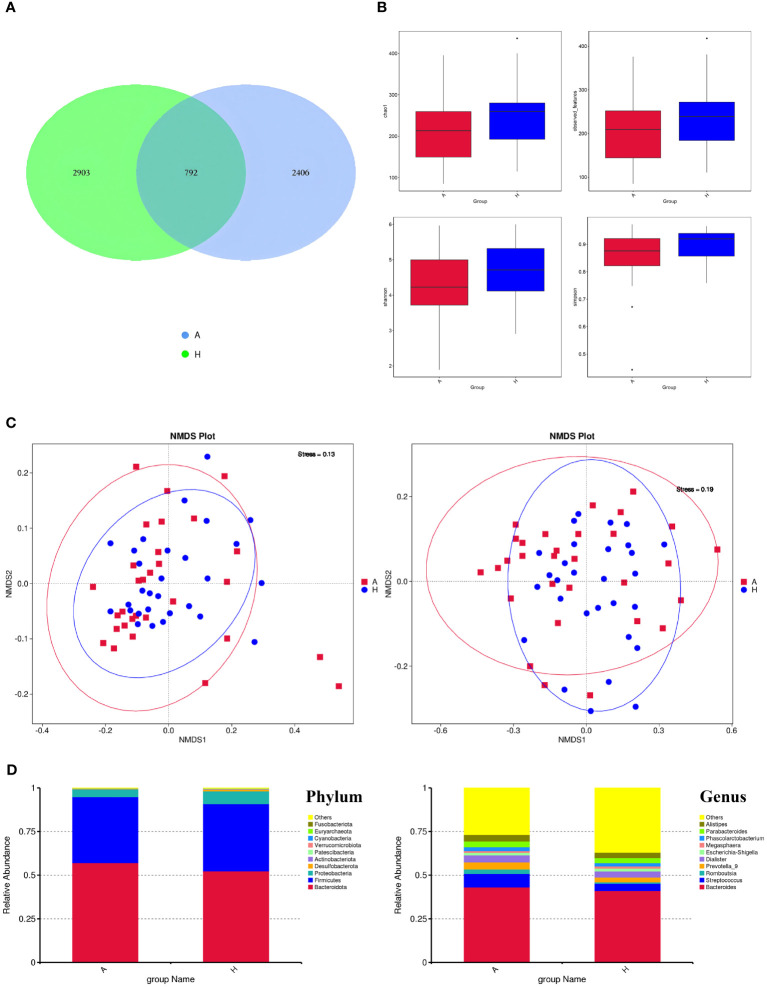
**(A)** The ASVs in the two groups (A is TAO, H is the healthy control group). **(B)** Alpha diversity comparison between the two groups. **(C)** NMDS plots (left: based on Weighted Unifrac distance; right: based on Unweighted Unifrac distance). **(D)** The comparison of intestinal flora at the Phylum level and Genus level.

We performed 16S rRNA gene sequencing of gut flora in both the TAO and healthy control groups, analyzing the shared and unique feature sequences between the two groups (**Figure 1A**). The analysis revealed 2406 characteristic sequences in patients with TAO and 2903 in healthy controls. Notably, patients with TAO exhibited lower characteristic sequence counts, suggesting reduced intestinal richness and diversity compared to healthy individuals. Subsequent assessments of gut microbiome diversity included α-diversity and β-diversity analyses. Evaluation of species richness indices (Observed_species and Chao1) and species diversity indices (Shannon and Simpson) did not yield significant differences between the two groups (**Figures 1B**). However, employing the non-metric multidimensional scaling method (NMDS) to analyze sample differences revealed distinct flora diversity between patients with TAO and healthy controls (**Figure 1C**). A stress value of less than 0.2 indicated an accurate reflection of sample dissimilarities by NMDS.

#### 3.2.2 Analysis of intestinal flora species composition

Following species annotation at various taxonomic levels, the top 10 species with the highest relative abundances at the phylum and genus levels were identified for each subgroup, with the remaining species categorized as “others.” The dominant intestinal flora composition in both patients with TAO and healthy controls exhibited similarity at the phylum level, with *Firmicutes*, *Bacteroidetes*, and *Proteobacteria* ranking highest in abundance. At the genus level, the top 10 dominant genera in the TAO group, in descending order of relative abundance, were *Bacteroides*, *Streptococcus*, *Prevotella*, *Dialister*, *Alistipes*, *Parabacteroides*, *Romboutsia*, *Phascolarctobacterium*, *Escherichia-Shigella*, and *Megasphaera* (**Figure 1D**).

#### 3.2.3 Differential analysis of intestinal flora

**Figure 2:**
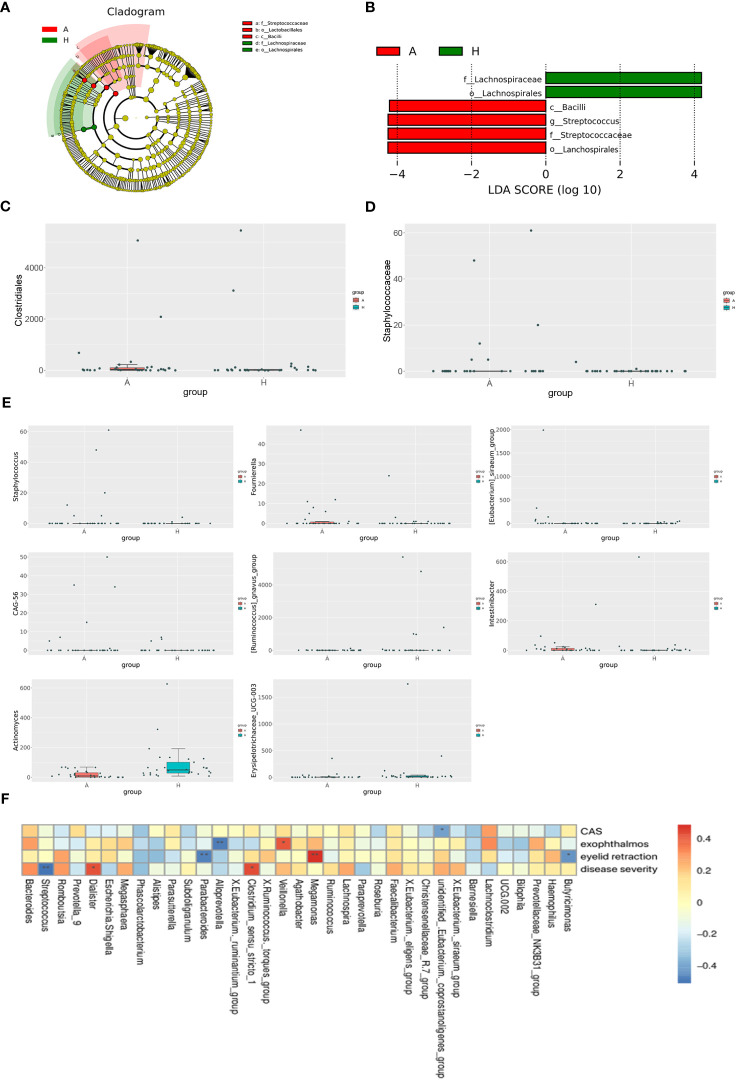
**(A)** The phylogenetic distribution of gut microbiota correlated with the two groups as shown by cladogram using LDA effect size analysis. **(B)** Histogram of LDA branch of gut dominant flora in two groups. **(C)** Analysis of species differences at the order level. **(D)** Analysis of species differences at the family level. **(E)** Analysis of species differences at the genus level. **(F)** Heatmap of correlation analysis between gut microflora and clinical indexes of TAO. *p ≤ 0.05; **p ≤ 0.01.

To elucidate differentially enriched intestinal flora in patients with TAO compared to healthy controls, a comparative analysis was conducted using LEfSe (**Figure 2A**). The results revealed the enrichment of four bacterial taxa in patients with TAO: *Bacilli*, *Lachnospirales*, *Streptococcaceae*, and *Streptococcus*. Conversely, two bacterial taxa were enriched in healthy controls: *Lachnospirales* and *Lachnospiraceae*. Branching maps at different levels were generated using LEfSe software (**Figure 2B**). These findings suggest that, aside from *lachnospirales*, which were co-enriched in both groups, other taxa could serve as biomarkers to differentiate between patients with TAO and healthy controls. Using the MetagenomeSeq method, species with significant differences between groups were identified. At the order level, *Clostridiales* differed significantly between the two groups (logFC>1 and P<0.05), while at the family level, Staphylococcaceae exhibited significant differences (logFC>1 and P<0.05). Additionally, at the genus level, Staphylococcus, Fournierella, *Eubacterium siraeum*, *CAG-56*, *Ruminococcus gnavus*, *Intestinibacter*, *Actinomyces* and *Erysipelotrichaceae UCG-003* exhibited significant differences (logFC>1 and P<0.05) between the two groups (**Figures 2C, D, E**).

#### 3.2.4 Correlation analysis between intestinal flora and clinical symptoms in patients with TAO

Spearman’s correlation coefficient was employed to investigate the relationships between clinical indicators of TAO, including active CAS score, disease severity, exophthalmos, eyelid recession, and the abundance of specific bacterial genera in patients with TAO. *Unidentified Eubacterium coprostanoligenes* exhibited a negative correlation with the CAS score, while *Veillonella* demonstrated a positive correlation with exophthalmos. Conversely, *Alloprevotella* showed a negative correlation with exophthalmos. Moreover, *Megamonas* displayed a positive correlation with the degree of eyelid recession, whereas *Parabacteroides* and *Butyricimonas* were negatively correlated with eyelid retraction. Additionally, *Dialister* and *Clostridium sensu stricto 1* exhibited positive correlations with disease severity, while *Streptococcus* showed a negative correlation with disease severity (**Figure 2F**).

### 3.3 Results of intestinal metabolites in patients with TAO and healthy control group

#### 3.3.1 Multivariate statistical analysis of intestinal metabolites

**Figure 3:**
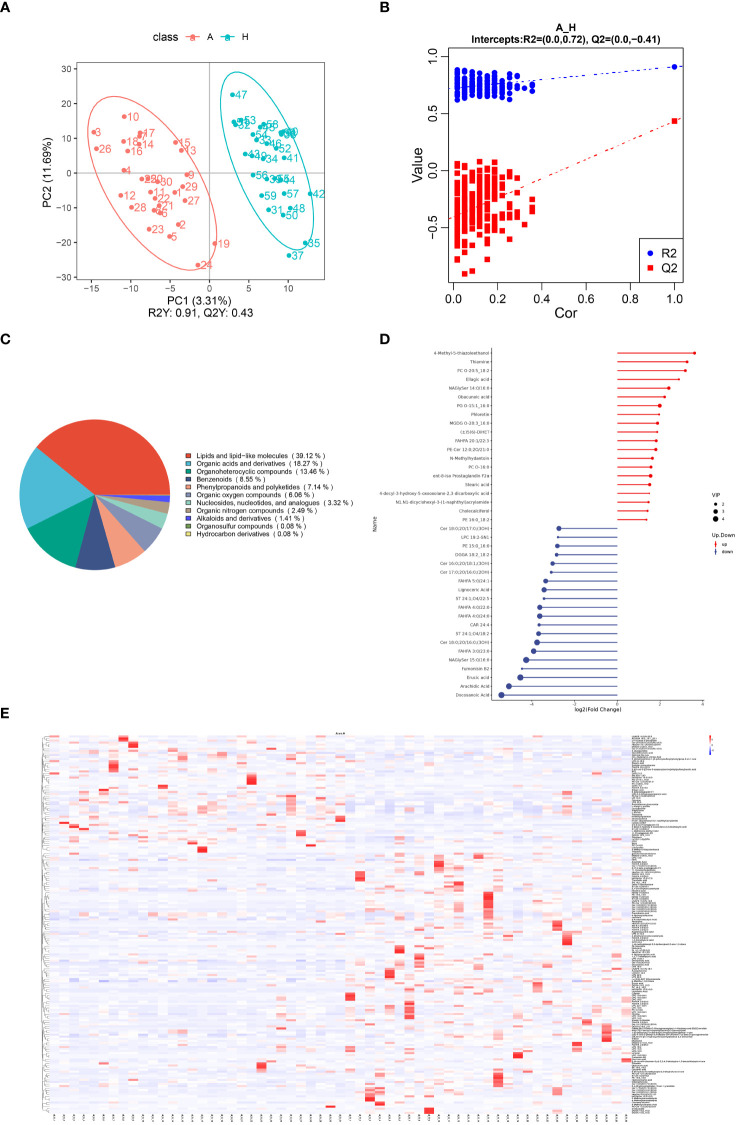
**(A)** Scatterplot of fecal metabolite PLS-DA scores for TAO and healthy control groups. **(B)** Equencing validation plot. **(C)** The classification of metabolic features. **(D)** Matchstick diagram of significantly altered metabolites (Blue dots represent downregulation, while red dots represent upregulation, with the length of rods indicating the magnitude of log2 (FC) and dot size representing the magnitude of VIP values). **(E)** Heat map analysis of fecal differential metabolites in TAO and healthy control groups (red indicating high expression and blue indicating low expression).

A total of 2234 metabolites were identified in fecal samples from patients with TAO and the control group. The scatter plot of PLS-DA scores showed a significant separation of metabolites between the two groups, indicating significant changes in fecal metabolites in patients with TAO (**Figure 3A**). After PLS-DA sorting validation, the model showed no signs of overfitting, as indicated by R2 data surpassing Q2 data and an intercept below 0 on the Q2 regression line (**Figure 3B**). These results demonstrate the efficacy of the PLS-DA model in distinguishing between sample groups, confirming its predictive ability and suitability for subsequent variance component analysis.

#### 3.3.2 Screening and identification of intestinal differential metabolites

Of the 2234 identified metabolites, 184 were significantly different between patients with TAO and healthy controls, with 63 upregulated and 121 downregulated. Chemical classifications of all identified intestinal metabolites were tallied, and pie charts were generated to illustrate the types and distribution of intestinal metabolites, revealing that 39.12% were enriched in lipids and lipid-like molecules (**Figure 3C**).

Further differentiation of fecal metabolites in patients with TAO involved screening for differential metabolites. The top 20 up- and down-regulated metabolites were selected for each differential metabolite ranking, represented in matchstick diagrams. The top 20 upregulated intestinal metabolites included compounds such as 4-Methyl-5-thiazoleethanol, Thiamine, PC O-20:5_18:2, Ellagic acid, NAGlySer 14:0/16:0, Obacunoic acid, PG O-15:1_16:0, Phloretin, MGDG O-28:3_16:0, (±)5(6)-DiHET, FAHFA 20:1/22:3, PE-Cer 12:0;2O/21:0, N-Methylhydantoin, PC O-16:0, ent-8-iso Prostaglandin F2α, Stearic acid, 4-decyl-3-hydroxy-5-oxooxolane-2,3-dicarboxylic acid, N1,N1-dicyclohexyl-3-(1-naphthyl)acrylamide, Cholecalciferol, and PE 16:0 _18:2. Notably, these metabolites were mainly enriched in lipid and lipid-like molecules, organic heterocyclic compounds, phenylpropanoids, and polyketides. Conversely, the top 20 down-regulated intestinal metabolites encompassed compounds such as Cer 18:0;2O/17:0;(3OH), LPC 19:2-SN1, PE 15:0_16:0, DGGA 18:2_18:2, Cer 16:0;2O/18:1;(3OH), Cer 17:0;2O/16:0;(2OH), FAHFA 5:0/24. 1, Lignoceric Acid, ST 24:1;O4/18:2, FAHFA 4:0/22:0, FAHFA 4:0/24:0, CAR 24:4, ST 24:1;O4/18:2, Cer 18:0;2O/16:0;(3OH), FAHFA 3:0/23:0, NAGlySer 15. 0/16:0, Fumonisin B2, Erucic acid, Arachidic Acid, and Docosanoic Acid. Notably, these metabolites were enriched in lipids, lipid-like molecules, and organic heterocyclic compounds (**Figure 3D**).

To visually illustrate the disparity in metabolite expression between the TAO and healthy control groups, a heatmap analysis was performed for significantly differentially expressed metabolites enriched in specific pathways. As shown in **Figure 3E**, colors indicate metabolite levels, and clear regions of high or low expression are discernible in both TAO and healthy populations, highlighting significant differences in the content of differential metabolites between the two groups. Notably, the identified significant differential metabolites can be used as markers to differentiate the two groups. They include ADGGA 18:3_16:1_22:1, HexCer 18:1;3O/24:0;(2OH), *Sorbitan monostearate* and so on (all AUC > 0.7) (**Supplementary Table 1**). These metabolites were clustered together based on their similar functions or involvement in shared metabolic pathways.

#### 3.3.3 Analysis of intestinal differential metabolite pathway enrichment

**Figure 4:**
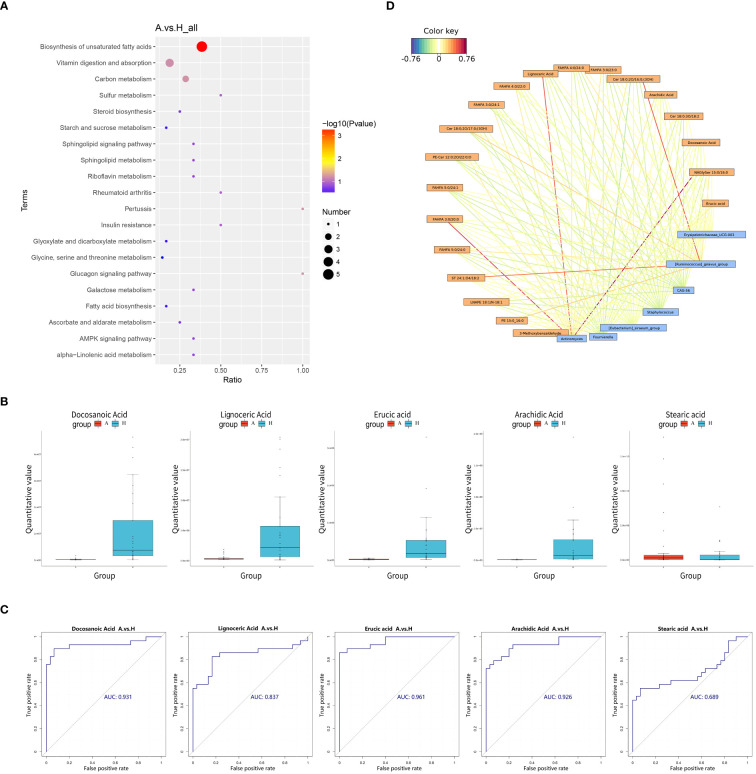
**(A)** Pathway analysis was visualized in bubble diagrams of TAO and healthy control groups (In these plots, circles indicate all matched metabolic pathways, with the color and size of the circles indicating the P-value of the metabolic pathway and the pathway impact value, respectively). **(B)** Comparison of Docosanoic Acid, Lignoceric Acid, Erucic Acid, Arachidic Acid, and Stearic Acid level between TAO group and control group. **(C)** ROC curve analysis of Docosanoic Acid, Lignoceric Acid, Erucic Acid, Arachidic Acid, Stearic Acid in TAO group and control group. **(D)** Relationship network between significantly altered microbes and metabolites (Blue boxes represent differential genera, yellow boxes represent differential metabolites, red color signifies positive correlation, and blue color signifies negative correlation).

Pathway enrichment analysis of the differential metabolites was performed using the KEGG database, and the bubble plots of the enriched KEGG pathways (only the top 20 results are shown) are shown in **Figure 4A**. The differential metabolites were found to be distributed among 30 metabolic pathways, with predominant enrichment observed in pathways such as biosynthesis of unsaturated fatty acids, the glucagon signaling pathway, pertussis, carbon metabolism, vitamin digestion and absorption, sulfur metabolism, insulin resistance, rheumatoid arthritis, galactose metabolism, and alpha-linolenic acid metabolism in. Notably, the unsaturated fatty acid biosynthesis pathway exhibited significance (P < 0.05), with five differential metabolites detected in this pathway: Docosanoic Acid, Lignoceric Acid, Erucic Acid, Arachidic Acid, and Stearic Acid.

Box plotting of five differential metabolites–Docosanoic Acid, Lignoceric Acid, Erucic acid, Arachidic Acid, and Stearic acid–enriched in the unsaturated fatty acid synthesis pathway showed that the quantitative values of intestinal metabolites enriched in unsaturated fatty acids were significantly lower in patients with TAO than in the healthy group, (**Figure 4B**). Subsequently, these five differentially expressed metabolites were further evaluated as potential biomarkers using ROC curves. The results showed that Docosanoic Acid, Lignoceric Acid, Erucic Acid, and Arachidic Acid could be used as biomarkers to differentiate between TAO and healthy groups, as illustrated in **Figure 4C**.

### 3.4 Joint multi-omics analysis

The 16S rRNA amplicon sequencing technology is an important tool for detecting the compositional structure of the gut flora. Metabolomics enables the measurement of metabolic alterations within a host ecosystem at a specific time point. To probe into the phenotypic shifts potentially induced by alterations in the host microbial community structure, a correlation analysis between the metabolome and microorganisms was conducted. A network diagram, based on the correlation coefficients of the differential genera and metabolites, was generated to illustrate the degree of correlation and association between these entities within the samples. Our findings revealed several noteworthy correlations: *Actinomyces* exhibited a positive correlation with NAGlySer 15:0/16:0, FAHFA 3:0/20:0, and Lignoceric Acid. Additionally, *Ruminococcus gnavus* demonstrated a positive correlation with Cer 18:0;2O/16:0;(3OH) and ST 24:1;O4/18:2 (**Figure 4D**).

## 4 Discussion

### 4.1 Gut flora characteristics in patients with TAO and healthy controls

The gut flora represents an important microbial ecosystem reflecting an individual’s health status, exerting a profound influence on the internal intestinal environment through dynamic interactions among diverse equilibriums (21). Our investigation into 30 patients with TAO and 29 healthy controls corroborated previous findings of reduced abundance and species diversity in the intestinal flora of patients with TAO compared to healthy controls (22, 23), aligning with studies indicating diminished gut microbial diversity across various diseases such as diabetes and cancer (24, 25). Thus, a decline in the diversity of gut microbes may be indicative of the severity of TAO. Additionally, our study demonstrated the dominance of *Firmicutes*, *Bacteroidetes*, and *Proteobacteria* at the phylum level and *Bacteroides* at the genus level in patients with TAO, consistent with previous research (22, 23, 26).

Furthermore, our investigation into the differences in bacterial flora between patients with TAO and healthy controls revealed notable findings. While no significant difference was observed between the two groups at the phylum level, a significant increase in the relative abundance of *Clostridiales* was identified in patients with TAO (logFC>1 and P<0.05). This finding aligns with previous multicenter studies associating *Clostridiales* with serum thyrotropin receptor antibody (TRAb) levels in patients with Graves’ disease and TAO (22). Although TRAb levels are significantly correlated with disease progression and prognosis (27), the precise role of *Clostridiales* in the pathogenesis of TAO remains uncertain. At the genus level, we observed a significant reduction in the abundance of *Ruminococcus* in patients with TAO patients compared to healthy controls (logFC>1 and P<0.05). Notably, the diminished level of *Ruminococcus gnavus*, a crucial intestinal bacterial genus, has been associated with various autoimmune diseases such as psoriasis and inflammatory bowel disease. Diminished levels of *Ruminococcus gnavus* have been shown to activate effector T cells, which are involved in autoimmune diseases (28, 29). Therefore, the reduction of *Ruminococcus gnavus* in patients with TAO may play a role in the development of the disease.

Rundle’s study on the natural course of the active phase of TAO concluded that the condition initiates with an autoimmune reaction persisting for several months, gradually intensifying over time. The pathological process characterized by orbital tissue inflammation, lymphocytic infiltration, glycosaminoglycan production, and edema is collectively termed the active phase of TAO, which is assessed using the CAS score to determine disease activity (30). In this study, we observed a negative correlation between *unidentified Eubacterium coprostanoligenes* and the CAS score, suggesting its association with the autoimmune response in TAO. Previous studies have indicated the involvement of *Eubacterium coprostanoligenes* in lipid homeostasis regulation (31). In patients with TAO, elevated inflammatory cytokine levels induced by hyperlipidemia may exacerbate ocular inflammation, leading to increased TAO activity (32, 33). In this study, we found a negative correlation between this bacterial group and disease activity, suggesting its potential role in regulating lipid homeostasis among patients with TAO, thereby reducing inflammatory cytokine levels and mitigating TAO activity. However, further studies are required to elucidate the mechanisms by which this bacterium regulates lipid homeostasis and its impact on TAO pathogenesis.

The degree of exophthalmos in patients with TAO is closely related to inflammation and the immune response. Throughout the disease progression, the release of inflammatory mediators triggers heightened activity in retrobulbar connective tissues and extraocular muscle fibroblasts, in patients with TAO, leading to the synthesis of abundant hydrophilic substances known as glycosaminoglycans. These substances, which possess water-absorbing properties, contribute to the hypertrophy of extraocular muscles. Additionally, orbital fibroblasts can differentiate into adipocytes, inducing adipose tissue hyperplasia. The combined effect of these processes elevates intraorbital pressure due to orbital restriction, ultimately resulting in exophthalmos (1). In this study, a positive correlation was observed between *Veillonella* abundance and exophthalmos. *Veillonella* is an important pathogen in oral diseases such as periodontitis, with its released metabolites capable of activating Toll-like receptors, thereby initiating inflammatory and immune responses (34, 35). In addition, *Veillonella* is enriched in the intestinal flora of patients with primary biliary cholangitis, an autoimmune disorder, suggesting its involvement in immune system development, particularly in infants (36, 37). Based on these findings, *Veillonella* may plays a role in the pathogenesis of exophthalmos in patients with TAO.

### 4.2 Intestinal metabolite profiles in patients with TAO and healthy controls

Few studies have investigated fecal metabolism in TAO samples. A European multicenter study reported enrichment of intestinal metabolites in short-chain fatty acids in patients with TAO without specifying related enrichment pathways (23). In the present study, we noted similarities between metabolic pathways in the feces of patients with TAO and those in orbital tissues, such as insulin resistance and β-alanine metabolism. This suggests the presence of an intestinal-ocular axis (9, 38, 39). Insulin resistance is closely related to the insulin-like growth factor 1 receptor (IGF-1R) (40). Insulin growth factor 1 (IGF-1) promotes the proliferation and differentiation of orbital adipose stem cells and fat accumulation. Notably, a cross-antigen exists between the thyroid-stimulating hormone receptor (TSHR) and IGF-1. Antibodies against TSHR can stimulate IGF-1 production, contributing to exophthalmos development. In addition, TSHR and IGF-1 form a functional complex that regulates cytokine expression, promoting hyaluronic acid accumulation, ocular inflammation, and extraocular muscle thickening, further exacerbating orbital fat accumulation (41, 42). The IGF-1R-induced accumulation of orbital fat in TAO may underlie insulin resistance, representing a potential molecular mechanism.

In our study, levels of intestinal metabolites enriched in the unsaturated fatty acid biosynthetic pathway were significantly lower in patients with TAO compared to healthy controls, suggesting impaired synthesis of unsaturated fatty acids in TAO. Unsaturated fatty acids play vital roles in antioxidation by neutralizing oxygen free radicals, thereby mitigating cellular damage (43, 44). Conversely, oxidative stress, which is prominent in TAO, promotes disease development by impacting orbital fibroblasts (45). KEGG pathway enrichment indicated blockage of the unsaturated fatty acid biosynthesis pathway, potentially may lead to a reduction in TAO’s antioxidant capacity and contribute to its pathogenesis.

### 4.3 Correlation analyses

Correlation analyses of gut microbes and their metabolomes provide insights into the interplay between the microbiome and gut metabolites. Alterations in gut flora abundance may affect gut metabolites. Our study identified a positive correlation between *Actinomyces* and NAGlySer 15:0/16:0, FAHFA 3:0/20:0 and Lignoceric Acid. Similarly, *Ruminococcus gnavus* positively correlated with Cer 18:0;2O/16:0;(3OH) and ST 24:1;O4/18:2. Notably, Lignoceric Acid participates in the unsaturated fatty acid metabolic pathway, suggesting *Actinomyce’s* involvement in this pathway. These findings underscore the intricate relationship between intestinal flora and metabolites in patients with TAO, shedding light on the synthesis of crucial bio signaling molecules. Such insights deepen our understanding of TAO pathogenesis and provide new ideas for therapeutic exploration.

### 4.4 Limitations and prospects

This study has certain limitations. First, we included only a limited number of patients for analysis, which may not fully represent the characteristics of the entire TAO patient population. To address this limitation, future studies could aim to expand the sample size and recruit more patients, thereby enhancing the reliability and accuracy of the results. This would contribute to a more comprehensive understanding of the characteristics and risk factors of intestinal flora and metabolites in patients with TAO. Second, certain confounding factors should be considered, and although every effort was made to minimize the differences in dietary habits and lifestyle between the two groups to reduce the influence of these factors on the experimental results, it was not possible to completely exclude all potential effects of diet and lifestyle on the intestinal flora and their metabolites. Patients’ dietary habits and lifestyles may influence gut flora and gut metabolites. For instance, staying up late and sleeping irregularly can disrupt our biological clock regulation mechanism, which regulates various physiological processes, including the balance of intestinal flora. Such irregularities may lead to imbalances in intestinal flora composition (46). Additionally, staying up late may affect changes in intestinal metabolites. Scientific evidence indicates that intestinal flora metabolites are crucial to human health, and maintaining regular sleep patterns is crucial for normal metabolite levels. Staying up late could induce abnormal changes in intestinal metabolites, such as an increase in inflammatory factors and accumulation of harmful metabolites, further exacerbating disease occurrences (47, 48). The effects of these factors on patients’ gut flora warrant further exploration in future studies.

## 5 Conclusion

In conclusion, this is the first study to analyze both the intestinal flora and metabolites in patients with TAO. Our findings unveil notable differences in the intestinal flora and metabolic profiles between patients with TAO and healthy controls, highlighting a disruption in intestinal metabolism, particularly in the biosynthetic pathways of unsaturated fatty acids among patients with TAO. By shedding light on these molecular intricacies, our study contributes to a deeper understanding of the pathogenesis of TAO, offering new insights into its prevention, diagnosis, and treatment strategies.

## Data availability statement

The datasets presented in this study can be found in online repositories. The names of the repository/repositories and accession number(s) can be found below: https://www.ncbi.nlm.nih.gov/, PRJNA1089481.

## Ethics statement

The studies involving humans were approved by Shanxi Eye Hospital Ethics Committee. The studies were conducted in accordance with the local legislation and institutional requirements. The participants provided their written informed consent to participate in this study.

## Author contributions

XZ: Writing – original draft, Data curation. KD: Writing – review & editing, Supervision. XZ: Writing – review & editing, Investigation. ZK: Writing – review & editing, Software. BS: Writing – review & editing.
