# Supplementary material for: Exploring gut microbiota and metabolite alterations in patients with thyroid-associated ophthalmopathy using high-throughput sequencing and untargeted metabolomics
Source: Front Endocrinol (Lausanne). 2024 Jul 29;15:1413890. doi: 10.3389/fendo.2024.1413890 (PMC11317416; doi:10.3389/fendo.2024.1413890)
Supplement: Supplementary file 1 [file Table_1.docx]

Supplementary Table 1 The identified significant differential metabolites

| ADGGA 18:3_16:1_22:1 |
| --- |
| HexCer 18:1;3O/24:0;(2OH) |
| Sorbitan monostearate |
| PG O-15:1_16:0 |
| PE-Cer 12:0;2O/21:0 |
| PC O-20:5_18:2 |
| LPA 22:6 |
| L-Alanyl-L-proline |
| ent-8-iso Prostaglandin F2 |
| DGDG 18:2_18:3 |
| Arachidic Acid |
| FAHFA 3:0/24:1 |
| HexCer 18:1;3O/16:0;(2OH) |
| FAHFA 5:0/24:0 |
| Delta-Tridecalactone |
| ST 24:1;O4/18:1 |
| NAGly 18:3/24:4 |
| PE-Cer 12:0;2O/22:0;O |
| Cer 18:0;2O/17:0;(3OH) |
| Cer 18:0;2O/16:0;(3OH) |
| Jasmone |
| Reserpine |
| FAHFA 3:0/23:0 |
| FAHFA 4:0/22:0 |
| FAHFA 4:0/24:0 |
| DL-Norvaline |
| Cer 18:0;3O/18:2 |
| Docosanoic Acid |
| LNAPE 18:1/N-18:1 |
| Acetophenone |
| LPS 22:6 |
| p-Mentha-1,3,8-triene |
| Erucic acid |
| DGGA 18:2_18:2 |
| NAGlySer 15:0/16:0 |
| Lignoceric Acid |
| LPC 18:3-SN1 |
| FAHFA 3:0/20:0 |
| FAHFA 4:0/20:0 |
| LPC 16:0-SN1 |
| Kinetin 9-riboside |
| FAHFA 5:0/24:1 |
| HexCer 18:1;3O/24:0;(2OH) |
| DGDG O-8:0_2:0、(3beta,9xi)-3-(beta-D-Glucopyranosyloxy)-14-hydroxycard-20(22)-enolide |
| ethyl 3-[(1-benzyl-4-piperidyl)amino]-2-cyanoacrylate |
| (2S)-4-Oxo-2-phenyl-3,4-dihydro-2H-chromen-7-yl beta-D-glucopyranosid |
| (2E,4E)-N-[2-(4-hydroxyphenyl)ethyl]dodeca-2,4-dienamide |
| Vitexin |
| Fumonisin B2 |
| PE-Cer 12:1;2O/22:0;O |
| ST 24:1;O4/18:2 |
| PE 15:0_16:0 |
| Cer 16:0;2O/18:1;(3OH) |
| HexCer 22:0;2O/12:1;O、3-Methoxybenzaldehyde |
| 4-Methoxybenzaldehyde |
| Cinnamyl alcohol |
| DGGA 18:2_18:2 |

(AUC > 0.7)
